# Functional neuroanatomy of mania

**DOI:** 10.1038/s41398-022-01786-4

**Published:** 2022-01-24

**Authors:** Gonçalo Cotovio, Albino J. Oliveira-Maia

**Affiliations:** 1 Champalimaud Research and Clinical Centre, Champalimaud Foundation, Lisbon, Portugal; 2 NOVA Medical School, NMS, Universidade Nova de Lisboa, Lisbon, Portugal; 3 Departamento de Psiquiatria e Saúde Mental, Centro Hospitalar de Lisboa Ocidental, Lisbon, Portugal

**Keywords:** Bipolar disorder, Neuroscience

## Abstract

Mania, the diagnostic hallmark of bipolar disorder, is an episodic disturbance of mood, sleep, behavior, and perception. Improved understanding of the neurobiology of mania is expected to allow for novel avenues to address current challenges in its diagnosis and treatment. Previous research focusing on the impairment of functional neuronal circuits and brain networks has resulted in heterogenous findings, possibly due to a focus on bipolar disorder and its several phases, rather than on the unique context of mania. Here we present a comprehensive overview of the evidence regarding the functional neuroanatomy of mania. Our interpretation of the best available evidence is consistent with a convergent model of lateralized circuit dysfunction in mania, with hypoactivity of the ventral prefrontal cortex in the right hemisphere, and hyperactivity of the amygdala, basal ganglia, and anterior cingulate cortex in the left hemisphere of the brain. Clarification of dysfunctional neuroanatomic substrates of mania may contribute not only to improve understanding of the neurobiology of bipolar disorder overall, but also highlights potential avenues for new circuit-based therapeutic approaches in the treatment of mania.

## Introduction

Mania is an episodic disturbance of mood, sleep, behavior, and perception. It is characterized by expansive, elated and/or irritable mood, increased energy, grandiosity, lack of sleep, impaired thinking, and poor judgment [1]. Importantly, the recurrence of such episodes has been identified as the diagnostic hallmark of bipolar disorder (BPD) and other bipolar spectrum disorders, estimated to affect 3–6% of the world population [2]. Due to its impact on patient functioning, commonly resulting in clinical, interpersonal, financial, and even legal consequences, mania is considered a very debilitating mental health condition, not only for patients suffering from this mood disorder but also for their family and friends [3].

Despite such relevant effects on patient well-being [4], diagnosis and treatment of mania is still a clinical challenge [5, 6], possibly due to a poor understanding of the underlying neurobiology [7, 8]. Different potential mechanisms for the pathophysiology of affective disorders, including mania, have been suggested. These include the dysregulation of synaptic neurotransmission, namely that of glutamate and its action on NMDA receptors [9, 10], which have also been shown to be promising treatment targets for affective disorders [11, 12]. On the other hand, dysregulation of synaptic plasticity, impacted at the molecular level by impaired function of microRNAs in translational regulation, has also been suggested as another potential mechanism [13]. Furthermore, several authors have proposed a neuroanatomical dysfunction substrate for mania pathophysiology, focusing on the impairment of functional neuronal circuits and brain networks (see Strakowski et al. for a comprehensive review of the functional neuroanatomy of BPD [14]). Nevertheless, previous clinical research has focused mainly on BPD, making the results difficult to interpret in the unique context of mania, with heterogenous and sometimes even contradictory findings. In fact, the cause for ambiguity in these findings may lay on the differences between trait- and state-dependent changes, which have been reported across several neuropsychiatric disorders [15], but may be particularly relevant in episodic mood disorders such as BPD. Here, some changes may occur only during an acute episode of mood disturbance and disappear once the symptoms remit (i.e., state) while other changes may be present during acute episodes as well as during euthymia (i.e., trait) [16]. Ultimately, the distinct clinical characteristics of mania, depression, mixed episodes, and euthymia observed in patients with BPD may be reflected also in differences in neuroimaging findings, contributing towards a lack of clarity regarding the unique functional neuroanatomy substrate of mania [16].

Disentangling the specificities of dysfunctional neuroanatomy in mania would be an invaluable contribution to the field. First, it would help to find state-dependent biomarkers that may assist in the differential diagnosis of mania, depression, and euthymia, and, most importantly, also in more challenging cases such as mixed affective episodes, subthreshold (hypo)mania, or latent bipolarity in unipolar depression [17]. Second, it would contribute to the overall understanding of BPD neurobiology and its neural pathophysiology. Finally, it may ultimately also guide research of specific innovative treatment strategies for mania [18, 19]. The current manuscript, rather than a systematic review of the current literature, is a comprehensive overview of the evidence regarding the functional neuroanatomy of mania. It aims to summarize the best available evidence, creating a convergent model for mania circuit dysfunction, while subsequently highlighting potential avenues for development of new therapeutic approaches for mania.

## Methods

The search was performed on MEDLINE/PubMed and Google Scholar between December 2020 and April 2021. Search terms were: “Functional” AND “Neuroanatomy” AND “Mania”. Articles in English, French, Portuguese, or Spanish were considered, regardless of the publication date or country of origin. To be considered in our review, articles had to report functional neuroimaging findings, namely functional magnetic resonance imaging (fMRI), positron emission tomography (PET) or single-photon emission computed tomography (SPECT), in patients with bipolar disorder, during a manic episode. Clinical trials, cohort studies, case-control studies, case series, systematic reviews, and meta-analysis were included. While evidence from single case reports was excluded, their reference lists were screened for additional articles, as were those from the included articles

### Disrupted functional brain networks in primary mania

Functional neuroanatomy of mania has been obtained from different neuroimaging studies, conducted mainly in patients with primary idiopathic mania, the majority of cases of mania, where a clear medical or toxic cause for the episode cannot be identified. The neuroimaging approaches used have mostly varied from fMRI to PET, taking advantage of several task-based and resting-state protocols [20–22]. Furthermore, different research designs have also been explored to clarify dysfunctional circuits associated with mania, through comparisons of patients with mania with healthy subjects, with the euthymic state, and/or with other neuropsychiatric syndromes, such as unipolar or bipolar depression [21, 22].

When considering the results from previously published meta-analyses addressing functional neuroimaging findings in mania, key limbic regions were shown to have impaired activity in patients with mania when compared to healthy volunteers. Specifically, Chen and colleagues, based on 8 studies, reported that in mania compared to healthy the inferior frontal gyrus, i.e., Brodmann Area (BA) 47, was hypoactive bilaterally, with a predominance on the right side, while the left thalamus was hyperactive [22]. Moreover, Hajek and colleagues, in a meta-analysis of 10 functional imaging studies involving response-inhibition paradigms, further confirmed that patients with mania showed lower activation not only in right inferior frontal gyrus (BA47) but also in the right medial frontal gyrus (BA9), and greater activation in the right insula (BA13) and bilateral basal ganglia [23]. Interestingly, in this meta-analysis, but not in Chen et al., lower activation of right inferior frontal gyrus (BA47), was found in bipolar disorder irrespective of mood state (euthymia or mania), suggesting that right inferior frontal gyrus hypoactivation should be further explored as a potential trait marker for bipolar disorder. Conversely, while Hajek et al. did not report significant changes in amygdalae, Chen et al. showed decreased activity of the right amygdala and increased activity of the left amygdala, alongside other meso-temporal structures such as parahippocampal gyrus and hippocampus, when considering jointly studies including mania and euthymia. Some reports of the functional neuroanatomy of mania were not included in these meta-analyses, possibly due to exclusion criteria related to experimental design, such as type of neuroimaging modality or even specific task paradigms. Additionally, since the publication of these meta-analyses, more studies reporting on functional neuroimaging findings in the context of mania have been published. For a more complete understanding of the functional neuroanatomy of mania, the results of these studies are also included in Table 1.

**Table 1:** Functional neuroimaging findings during primary mania episode.

Article	Region	Side	Activity	Control	Study	Task
Abler et al. 2008	NAc.	Bilateral	↓	HS	fMRI	Reward task
Abler et al. 2008	NAc.	Bilateral	≈	Schiz.	fMRI	Reward task
Alonso‐Lana et al. 2019	VMPFC DLPFC/PC (BA6) Parietal cortex/superior precuneus	Bilateral Left Bilateral	≈		fMRI	Working memory task
			↓	Euth.^a^		
			↓			
Alonso‐Lana et al. 2019	VMPFC DLPFC/PC (BA6) Parietal cortex/superior precuneus	Bilateral Left Bilateral	↑		fMRI	Working memory task
			↓	HS		
			↓			
Altshuler et al. 2005a	Amygdala Lateral OFC	Left Bilateral	↑ ↓	HS	fMRI	Affect-laden task
Altshuler et al. 2005b	Lateral OFC (BA47) Hippocampus Cingulate cortex (BA24)	Right Right Left	↓	HS	fMRI	Go-No Go task
			↓			
			↓			
Bermpohl et al. 2009	Amygdala	Left	↑	HS	fMRI	Affect-laden task
Bermpohl et al. 2010^b^	Lateral OFC Lateral OFC Ventral striatum PCC	Left Left ND Right	↑^c^ ↓^d^ ≈ ↓	HS	fMRI	Reward task
Blumberg et al. 1999	MFG (BA10) OFC (BA11)	Right Right	↓ ↓	Euth.^e^	PET	Word generation task
Blumberg et al. 1999	MFG (BA10)	Right	↓	HS	PET	Word generation task
Blumberg et al. 1999	OFC	Bilateral	↓	HS	PET	Resting state
Blumberg et al. 2000	Dorsal ACC Head of caudate	Left Left	↑ ↑	Euth.^e^	PET	Resting state
Caligiuri et al. 2003	Globus pallidus Thalamus Caudate	Left Right Right	↑ ↓ ↓	BD^e^	fMRI	Reaction-time task
Caligiuri et al. 2003	SMA (BA6) Globus pallidus PMA (BA4)	Left Left Right	↑ ↑ ↓	HS	fMRI	Reaction-time task
Chen et al. 2010	Amygdala Hippocampus	Right Right	↓ ↓	Euth.^a^	fMRI	Affect-laden task
Chen et al. 2010	OFC Caudate	Right Left	↑ ↓	HS	fMRI	Affect-laden task
Drevets et al. 1997^f^	Subgenual prefrontal cortex	ND	↑	HS	PET	Resting state
Foland et al. 2008	Amygdala VLPFC (BA47)	Left Bilateral	↑ ↓	HS	fMRI	Affect-laden task
Lennox et al. 2004	Insula PCC Amygdala Subgenual ACC	Left ND Bilateral ND	↑ ↑ ↓ ↓	HS	fMRI	Affect-laden task
Liu et al. 2012	Rostral prefrontal cortex (BA10)	Right	↓	HS	fMRI	Affect-laden task
Mazzola-Pomietto et al. 2009	VLPFC	Bilateral	↓	HS	fMRI	Go-No Go task
Rubinsztein et al. 2001	Dorsal ACC (BA32) VMPFC (BA10) IFG (BA47)	Left Right Bilateral	↑ ↓ ↓	HS	PET	Decision-Making task
Strakowski et al. 2008	PCC (BA23, BA29) ACC (BA32) Thalamus Precuneus (BA7, BA39) Middle temporal gyrus (BA21, BA37)	Bilateral Left Left Left Left	↑ ↓ ↓ ↓ ↓	HS	fMRI	Response-inhibition task
Strakowski et al. 2011	Amygdala Fusiform VLPFC ACC Cerebellar vermis Parahippocampus IFG IFG Putamen Lingual gyrus Medial thalamus MFG SFG PC Precuneus	Right Left Bilateral Right ND Right Right Left Right Right ND Bilateral Right Right Right	↓↑^g^ ↓↑^g^ ↓ ↓ ↓ ↓↑ ^g^ ↓↑^g^ ↓ ↓↑ ^g^ ↓ ↓ ↓ ↓↑^g^ ↑ ↓	HS	fMRI	Affect-laden task

≈ – similar, ↑ = increase, ↓ = reduced, *ACC* anterior cingulate cortex, *BA* brodmann area, *BD* bipolar depression, *DLPFC* dorsolateral prefrontal cortex, *Euth*. euthymic state, *fMRI* functional magnetic resonance imaging, *HS* healthy subjects, *IFG* inferior frontal gyrus, *MFG* middle frontal gyrus, *NAc*. nucleus accumbens, *ND* not defined, *OFC* orbitofrontal cortex, *PC* precentral cortex, *PCC* posterior cingulate cortex, *PET* positron emission tomography, *PMA* primary motor area, *Schiz*. schizophrenia, *SFG* superior frontal gyrus, *VLPFC* ventrolateral prefrontal cortex, *VMPFC* ventromedial prefrontal cortex.

^a^Within-subject analysis.

^b^After symptoms remission no differences were found between manic remitted patients and healthy subjects in OFC activity.

^c^During expectation of increasing gain in the reward task.

^d^During expectation of increasing loss in the reward task.

^e^Between-subject analysis.

^f^Unipolar and bipolar depressed patients had decreased activation in subgenual prefrontal cortex relative to healthy subjects.

^g^Overall resulting activity differ according to the analysis performed and/or to the specific fMRI task cue.

One of the most consistent findings in the functional neuroanatomy of mania has been hyperactivity of the left amygdala in response to emotional cues, observed when comparing mania with healthy in fMRI studies [24–26], and also found to correlate with severity of mania symptoms [25]. However, when comparing mania with euthymic state in bipolar disorder, left amygdala hyperactivation was not found and, rather, decreased activation of the right amygdala was observed [27]. Together, these findings support the hypothesis that activity imbalance between left (elevated) and right (reduced) amygdalae is an important functional neuroimaging correlate of mania [28]. However, it is important to also consider that another study has shown a bilateral reduction in amygdala activity in mania relative to healthy subjects [29], and others have failed to show any pattern of differences in amygdala activation in mania relative to healthy subjects [14, 30]. Nevertheless, it would be interesting to explore, across studies, potential differences in the right-left imbalance of amygdala activity between patients with mania and healthy volunteers. Importantly, fMRI findings in other meso-temporal structures support the hypothesis of lateralized imbalance of activity, with decreased activity in the right hippocampus and parahippocampal gyrus of patients with mania, during cognitive and emotion-associated tasks, when comparing with healthy subjects and to euthymia [27, 31]. Overall, these findings support that activity in the amygdala and other meso-temporal hubs of emotional networks in the limbic system [16, 32–34] is dysregulated in mania, with most studies pointing towards increased activity in the left hemisphere or decreased activity on the right.

**Fig. 1:**
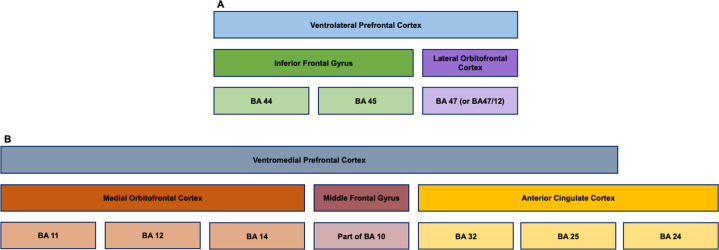
Neuroanatomy of ventrolateral and ventromedial prefrontal cortices. While there is variability in the nomenclature and in the organization of ventrolateral (**A**) and ventromedial prefrontal cortices (**B**), this diagram represents previously suggested models [36, 40–45] for the neuroanatomy of these structures, focusing on regions and areas that are critical for interpretation of the functional neuroanatomy of mania.

The prefrontal cortex (PFC) has also been consistently reported as a key region for the regulation of emotional behavior [32], and its dysfunction has been associated with mood disorders [35]. Noteworthy, the PFC is a complex structure [36] that, in addition to contributing to emotional regulation, has also been associated with different cognitive functions, such as working memory, attention, reward appraisal, and decision-making, functions that are supported by multiple reciprocal connections to distinct brain regions [36, 37]. Probably due to such multi-domain functions, while PFC has been persistently implicated in the functional neuroanatomy of mania, its specific role has been difficult to interpret [38]. Nevertheless, in fMRI and PET studies, assessing response to emotional stimuli [24, 26, 27, 39], response-inhibition tasks [31], and decision-making paradigms [40], the most consistent findings in mania relative to healthy subjects have been reduced activation on the right side of the brain or bilaterally in the ventrolateral prefrontal cortex (VLPFC), a brain region including the lateral orbitofrontal cortex [lOFC, also designated as Brodmann area (BA) 47 or BA47/12 due to correspondence of human BA47 to monkey BA12 [36, 41–44]; also see Fig. 1]. Similarly, in mania relative to healthy subjects, reduced activation has also been described in the right ventromedial prefrontal cortex (vmPFC), a heterogenous brain region that includes parts of the anterior cingulate cortex (ACC), namely BA25 and BA32, the middle frontal gyrus, namely BA10, and regions of the medial OFC (mOFC), such as BA11, BA12, and BA14 [36, 40, 44, 45] (Fig. 1). Interestingly, in a PET study, hypoactivity of the right BA10 and right BA11, areas included in VMPFC, was also observed during a word generation task when patients with mania were compared to euthymic state [46]. Moreover, in the same study, hypoactivity of the right BA10 and bilateral OFC, during a word generation task and in resting state, respectively, was also detected when patients with mania were compared to healthy subjects [46]. These results further support that under-activation of these regions may be a neuroimaging biomarker of mania. However, in what possibly reflects the complex roles of the PFC in human behavior, in another study, when compared to healthy subjects, patients with mania had both decreased and increased activation of left VMPFC and VLPFC, during gain and loss expectation states, respectively, in a reward decision-making task [47]. Furthermore, in fMRI during a working memory task, patients with mania had increased activation of the VMPFC bilaterally, relative to healthy subjects, while the activity of the left dorsolateral prefrontal cortex (DLPFC) was reduced [48]. Considering all the evidence above, it seems that the PFC is hypoactive in patients with mania, particularly in the right hemisphere, in certain cognitive and decision-making contexts.

Another region of interest that has been reported to be impaired in mood disorders [35, 49], including mania [35], is the cingulate cortex, particularly the anterior cingulate cortex (ACC), also designated as BA24, 25, and 32 [50] (Fig. 1). In fact, these brain regions have been considered part of the VMPFC, and their role has been implicated in several functions related to the PFC [45]. Hence, as is the case for the prefrontal cortex, a role for the ACC within a global vision for the functional neuroanatomy of mania has been difficult to establish [38]. When comparing patients with mania and healthy controls in fMRI studies, the former had reduced activation of the ACC in a Go-No Go task [31] and in response to emotional cues [29], but increased activation in working memory [48] and response-inhibition tasks [51]. On the other hand, in PET studies, patients with mania had increased activation of left ACC in resting state [35] and during a decision-making task [40], relative to healthy subjects, as well as when compared to euthymia [52]. Importantly, in patients with both unipolar and bipolar depression, ACC activity was found to be decreased in similar conditions [35]. Moreover, as described above for the left amygdala [25], there is a positive association between left ACC activity and severity of mania symptoms [40]. Thus, there is evidence for left-sided hyperactivity of the ACC in mania, while in certain conditions this area can be inappropriately hypoactivated.

Several subcortical structures have also been implicated in mania circuit dysfunction, the basal ganglia in particular [14, 22, 38]. Patients with mania had increased activity in the left basal ganglia, namely the globus pallidus and caudate, in a reaction-time task [53] and in response to affective simuli [27], when compared to healthy subjects, as well as bipolar depression [53] and euthymia [52]. Interestingly, and similar to what was described above for lateralized dysfunction of the amygdala, reduced activation of the right caudate in a reaction-time task was found in mania when compared to bipolar depression [53]. Nevertheless, the available evidence is mostly supportive of hyperactivity of the left basal ganglia in mania, supporting the importance of such structures in the regulation of emotional behavior [54, 55]. While other cortical and subcortical regions have also been implicated in the functional neuroanatomy of mania, these findings have not been consistently replicated, and cannot be appropriately interpreted in the current state of the art. Among others, they include not only increased activation of left supplementary motor area [53], left insula [29], and bilateral posterior cingulate [29] but also reduced activation of right primary motor cortex [53], right posterior cingulate [47], supplementary motor area [48], bilateral parietal cortex [48], bilateral [48] or left [51] precuneus, left middle temporal gyrus [51], left [51] and right [53] thalamus, and bilateral nucleus accumbens [56].

Noteworthy, three studies reported functional neuroimaging changes before and after mania treatment, i.e., in mania vs. euthymic state, using within-subject analyses. Two of these studies reported bilateral VMPFC (BA11) hyperactivity that remained after treatment [27, 48], suggesting that this may be a potential trait biomarker for bipolar disorder. These findings are inconsistent with the third longitudinal study reporting that, in a reward decision-making task, patients with mania no longer showed activity changes in left VMPFC and VLPFC after symptom remission [47]. Such heterogeneity of results supports a complex role for the VMPFC in the functional neuroanatomy of mania, further supported by the fact that, in response to certain stimuli, BA10/BA11 is hypoactive in patients with mania compared to a different group of euthymic patients, using between-subject analyses [46], while in response to other stimuli BA11 is inappropriately activated in patients with bipolar disorder, irrespective of mood state [27, 48]. These longitudinal studies have also suggested candidates for state markers of mania, that were not observed when patients entered euthymia. In fact, in comparisons to euthymic state, patients during mania were reported to have decreased activation in left DLPFC/precentral cortex (BA6) and bilateral superior parietal cortex/precuneus (BA7) by some authors [48], or in the right hippocampus and right amygdala by others [27]. Moreover, considering that left amygdala hyperactivity is a consistent finding in mania [24–26], it is also reasonable to consider that imbalanced activity between the left (elevated) and right (reduced) amygdalae may be a more accurate correlate of mania [28]. Further and better powered longitudinal studies, with experimental designs explicitly assessing differences in activity between the right and left hemispheres, are necessary to clarify potential roles for the activity of these brain regions as state markers of mania, or trait markers of bipolar disorder, ideally while resolving conflicting findings from previous studies [27, 46]

**Fig. 2:**
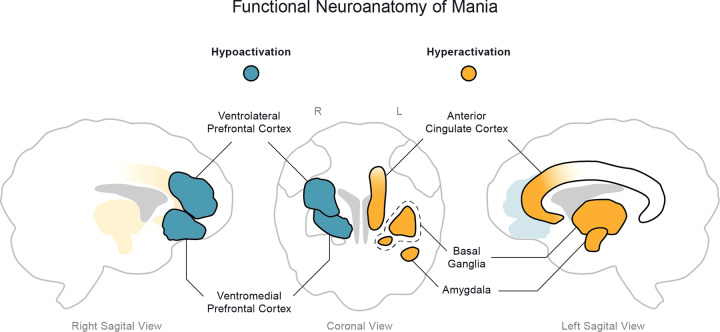
Functional neuroanatomy of mania. Evidence from different functional neuroimaging studies suggest that there is a reduced activity of right ventromedial and ventrolateral prefrontal cortices and an increased activity of left amygdala, left anterior cingulate cortex, and left basal ganglia in mania.

From the evidence presented above, in meta-analyses as well as several of the individual original studies, we hypothesize that mania is characterized by lateralized dysfunction of the limbic system, mainly involving the left amygdala, right (or bilateral) ventral PFC, left ACC, and left basal ganglia (Fig. 2). Not surprisingly, reciprocal connections between many of these regions, in particular the amygdala and PFC, have been shown to be disrupted. Relative to healthy controls, in patients with mania, the left amygdala was more negatively connected to the left ACC and right VMPFC [26], but less connected to the left caudate and putamen [57], and left [57] or bilateral [26] VLPFC, while the right amygdala was more connected to the right hippocampus [57]. Interestingly, negative connectivity from the left amygdala to bilateral VLPFC was negatively associated with mania symptom severity [26]. Moreover, in patients with mania, both amygdalae were less connected not only to the ACC in comparisons with euthymic patients with bipolar disorder [58], but also to the VLPFC and striatum in comparisons with healthy controls [57]. Finally, within PFC regions, patients with mania had increased positive connectivity between the right VLPFC and bilateral medial PFC, and reduced negative connectivity between bilateral medial PFC and bilateral DLPFC, when compared to healthy subjects and patients with schizophrenia [59], while the ACC was less connected to the left OFC in comparisons with healthy volunteers [60]. There is evidence that the prefrontal cortex modulates amygdala and striatal responses to emotional contexts and cues [61, 62], the net result of which has been associated with emotional behavior correlates [63], and with disruption of these connections possibly associated with affective disorders [26]. We thus propose that, during mania, a disconnection between the amygdala and prefrontal cortex [26, 32] may lead to hypoactivation of the ventral PFC, on the right hemisphere or bilaterally. Lateralized hypoactivity of the PFC may ultimately fail to modulate left amygdala activity, generating overt hyperactivity of left-sided limbic structures such as the ACC and basal ganglia, which also have impaired connections with the amygdala [26, 57, 58]. Nevertheless, as has been previously supported by Wang and colleagues [64], future studies focusing specifically on functional connectivity patterns in mania and bipolar disorder may contribute towards identifying biomarkers for these conditions, while clarifying the role of these connections in the neurobiology of emotions.

Interestingly, and while the development of adequate animal models of mania has been challenging [65–68], our hypothesis is further supported by evidence obtained in animal work. In proxies of affective and cognitive features of mania, such as response inhibition, impulsivity, and hedonic state assessed by sucrose preference [67, 69, 70], the PFC, namely OFC and ACC, as well as the amygdala, have been consistently implicated. When these brain regions are disturbed, animals have shown mania-like behavior, such as impulsivity, increased motor activity, or increased goal-directed activity [71–76]. For example, amygdala lesions were associated to greater impulsivity while OFC lesions had the opposite effect, increasing preference for larger but delayed rewards [72]. Such findings suggest that OFC is involved in evaluating the incentive value of outcomes [72] and disrupting/modulating its activity was associated with impaired response-inhibition and impulsive behavior [71]. Moreover, other regions have also been associated with persistent impulsive behavior when lesioned in animal models, such as nucleus accumbens core [77, 78], which has also been reported to be dysfunctional in mania [56]. Interestingly, mania has also been associated to reward appraisal disturbances and impaired response inhibition, which may explain the occurrence of disinhibition and increased goal-directed activity observed in patients during acute manic episodes [56]. Future research in animal models assessing different behavior and cognitive constructs relevant to mania, such as reward responsiveness, reward learning, reward valuation, cognitive control, or social communication, according to the Research Domain Criteria (RDoC) framework [79], may provide further insight in the functional neuroanatomy of mania. While most studies including animal models do not focus on the association of specific cognitive functions with lateralized brain regions, one demonstrated that right-sided brain lesions were predominantly associated with hyperactivity, a symptom also observed in mania [76]. Noteworthy, the proposed model of lateralized dysconnectivity and dysfunction in mania also aligns with the mood laterality theory, a classical vision of the neurobiology of emotions [80, 81]. This theory suggests that emotional functions are lateralized in the brain, with negative and positive emotions associated with the function of the right and left brain hemispheres, respectively [80]. For example, when studying food-reward processing in healthy volunteers, activation of both the amygdala and orbitofrontal cortex in the left hemisphere was observed as a response to highly motivating incentives [82]. Interestingly, mania has been associated with an elevation of achievement motivation [8], and the mood laterality theory is thus consistent with left-side overactivity [24–26] and right-sided impairment [31, 83–87] in mania. Moreover, it is also in agreement with structural findings in neuroimaging studies of bipolar disorder (for a comprehensive review please see Blond et al. 2012 [16]).

Contrary to structural neuroimaging, which may only reveal static and/or overt neuroanatomic changes, the functional studies reviewed here have the advantage of offering an opportunity to clarify more complex and dynamic brain alterations. Nevertheless, there are potential limitations that should be considered. Functional MRI does not truly provide a measure of brain metabolism. Instead, it relies on a blood-oxygen-level-dependent (BOLD) signal, an indirect measure of activity that is obtained in different contexts, tasks, and/or time-points [20]. Depending on the design, fMRI studies can lead to inconsistent findings due to the impact of tasks [14] and/or reliability of BOLD signals [88]. On the other hand, despite being a closer reflection of brain metabolism, PET has low spatial resolution and, given the use of ionizing radiation, may not be applied multiple times [20, 89]. These potential limitations highlight that additional research strategies merit consideration to help clarify the functional neuroanatomy of mania.

### From anatomy to function in lesional mania

While most mania episodes occur in the context of primary idiopathic BPD, patients may develop manic symptoms secondary to organic insults such as brain lesions, in what is typically designated as lesional mania. Common causes of lesional mania include stroke, traumatic brain injury, or tumors [90]. While several clinical features may signal the possibility of lesional mania, distinguishing this condition from primary BPD is a clinical challenge [91], and the similarity of their clinical profiles has been used to suggest that studying lesional mania can be an important approach to understand the functional neuroanatomy of mania [92]. In fact, in the context where brain lesions occur prior to mania, the association between brain structure change and clinical presentation is much clearer, allowing to highlight networks that may be missed in functional neuroimaging studies, or resolve conflicting findings [91, 93]. Importantly, one should be mindful that lesion locations, in the vast majority of cases, can be interpreted as equivalent to inactive or hypoactive brain regions [91].

In one of the first and largest case series of lesional mania, lesions were commonly located in the right thalamus, right caudate, right orbitofrontal, and right basotemporal cortices, while in cases of lesional depression the affected areas were more widely distributed on the left hemisphere [94]. A later study with a smaller case series reported similar findings, additionally showing that there was a decrease in ^18^Fluorodeoxyglucose uptake in right basotemporal and right superior frontal areas, even when those regions were not directly insulted [95]. These results led to the hypothesis that mania may be associated with a functional circuit encompassing the orbitofrontal cortex, basotemporal cortex, and basal ganglia [81, 83, 94–102]. It is noteworthy that these regions overlap with functional neuroimaging findings in primary mania described above. More recently, we systematically revised the available literature on lesional mania, and performed pooled analyses of all published cases [92]. We confirmed that lesions causing mania were over-represented in the right relative to the left hemisphere, namely in several mesiotemporal and temporal regions, as well as the basal ganglia and thalamus. Given the predominance of lesions in the right hemisphere, additional comparisons were performed with a control cohort of right hemisphere lesions [103], to confirm that the topography of lesional mania is not biased by the normative distribution of brain lesions. In addition to mesiotemporal and temporal regions and the thalamus, patients with lesional mania had larger lesions in ventral areas of the prefrontal cortex, with the latter affected bilaterally rather than predominantly on the right side. Interestingly, while the basal ganglia structures have been consistently reported to be associated with lesional mania [91], these regions were not identified in the comparisons with control lesions. Nevertheless, our findings support that lesions leading to mania are not randomly distributed, and preferentially affect hubs of the mania circuit proposed above [81, 83, 94–102].

While localization-based lesional studies have shed light on the functional neuroanatomy of mania, this methodology has important limitations. Most importantly, entirely non-overlapping lesion locations can be associated with the same clinical syndrome, a constraint that has been observed in several lesional neuropsychiatric syndromes [104, 105], including mania [92]. In fact, as suggested previously [95], the emergence of a clinical syndrome can result from physiological changes in distant regions preserved from the original lesion, but nevertheless connected to the lesion location, a phenomenon known as diaschisis [106]. Lesion network mapping is a recently developed neuroimaging methodology, developed to address this phenomenon [107], that takes advantageous of normative connectomes [108, 109] to highlight regions that are functionally connected to each lesion location, thus resulting in brain network maps. Two studies have applied this approach to lesional mania [110, 111]. Lee and colleagues showed that the temporal lobe and OFC, on both sides of the brain, were more functionally connected to lesion locations associated with mania than control lesions, with the possibility of negative connectivity with the DLPFC, also bilaterally, suggested. In the same study, exploring these findings in independent cohorts of primary bipolar disorder patients, the authors compared mania and euthymia to show that the temporal lobes had decreased connectivity to the VLPFC, and to suggest that the left amygdala may have increased connectivity with the DLPFC, bilaterally [110]. We participated in another study, with a larger connectome and larger mania and control cohorts, in order to improve the specificity of the resulting lesional mania connectivity map. Across multiple lesion cohorts, we found that lesion locations associated with mania, when compared to control lesions, were more connected to the right OFC (VLPFC; BA47), right inferior temporal gyrus, and right frontal pole (VMPFC; BA11) [111].

The evidence across lesional mania studies further supports our hypothesis of lateralized functional disruption of the limbic system in mania (Fig. 2). The findings of direct lesions, or potential indirect effects of lesions, affecting, among others, mesiotemporal and ventral prefrontal areas to cause mania [92, 110, 111], highlight the importance of left amygdala overactivation and right ventral PFC hypoactivation associated with primary mania. Nevertheless, it is important to consider that, when interpreting the results of lesional studies, one should consider that these approaches ignore post-lesional neuronal tissue remodeling and cerebral dynamic recovery processes. Moreover, lesions may simultaneously damage the brain cortex and fibers of passage making it difficult to accurately determine if the deficit results from damaging cortical regions, fibers of passage, or both. Again, these underline that a comprehensive understanding of neuroimaging findings across study methodologies is fundamental when describing the functional neuroanatomic substrate of a neuropsychiatric disorder.

### Functional neuroanatomy of mania as a target of therapeutic neuromodulation

**Fig. 3:**
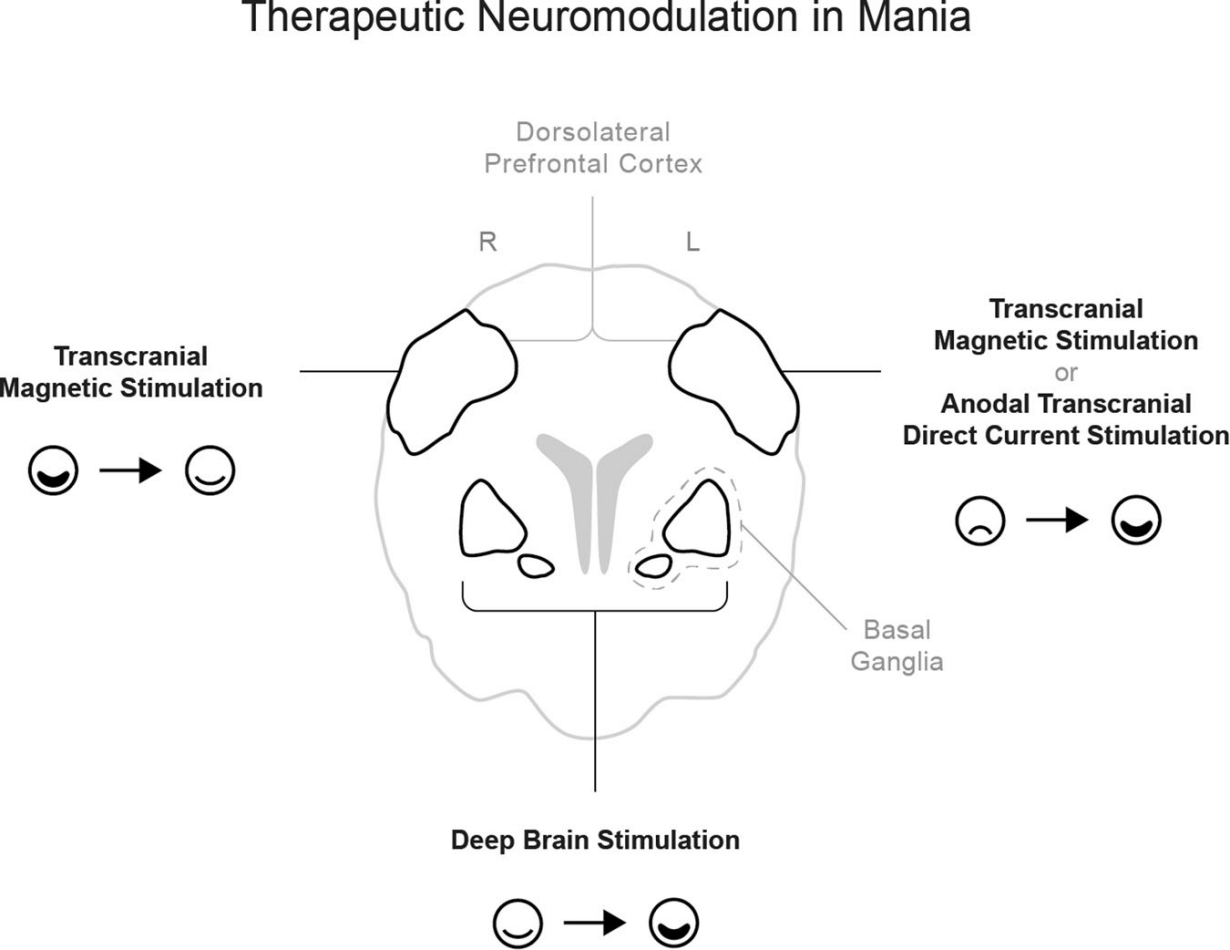
Therapeutic neuromodulation in mania. The functional circuit of mania may help define potential therapeutic neuromodulation targets to treat or avoid mania.

The ultimate goal of exploring and formulating a functional neuroanatomic and neurobiological model of mania is its potential use in guiding new treatment strategies (Fig. 3). Specifically, the model we have proposed above for circuit dysfunction in mania i.e., reduced activity of right ventral PFC and increased activity of the left amygdala, left ACC, and left basal ganglia, may support the use of these critical hubs as potential targets for therapeutic brain stimulation strategies (Fig. 2). In fact, as described in this section, the dysfunctional brain network of mania proposed above aligns with previously reported effects of non-invasive brain stimulation on mania symptoms.

High frequency repetitive transcranial magnetic stimulation (HF-rTMS), thought to positively modulate the targeted regions [112, 113] has shown beneficial effects in mania, when applied to the right DLPFC [18, 114–116]. On the other hand, HF-rTMS and anodal transcranial direct stimulation (a-tDCS) of the left DLPFC, both used to treat episodes of major depression [117], may result in manic symptoms as side effects [118–124]. Since DLPFC is functionally connected to VMPFC, and specifically the ACC [125–127], it may be an “entry point” to the dysfunctional mania circuit described above, particularly given the lateralization of pro-manic and anti-manic effects of focal DLPFC stimulation. In our own previous work, we have explored the potential functional impact of non-invasive brain stimulation treatment strategies considering the lesional mania network map developed when comparing mania with control lesions [111]. Consistently with clinical effects, we found that the left and right DLPFC had opposite connectivity to this map, which includes the right VLPFC (OFC; BA47) and the right VMPFC (frontal pole; BA11). When considered jointly, the findings mentioned above further support the hypothesis that modulation of DLPFC activity impacts VLPFC and VMPFC function. Hence, if treatment targets in DLPFC are optimized, supported by the functional neuroanatomy model of mania, enhanced efficacy and/or fewer side effects may be obtained in neuromodulatory treatment of affective disorders, including mania. Furthermore, in our previous work, optimal targets were additionally suggested for future use in non-invasive brain stimulation trials for mania, not only both in right (MNI 20, 36, 52) and left (MNI −20, 2, 64 and −36, 44, 38) prefrontal cortices but also right orbitofrontal cortex (MNI 42, 42, −20) [111]. Exploring new rTMS treatment targets will certainly have significant clinical implications. In addition to relief of core mania symptoms [18, 114–116], the proposed anti-suicidal effects of rTMS [128] may contribute towards avoiding suicide in the context of bipolar disorder [129].

Mania has also been reported as a side effect of deep brain stimulation (DBS), a different strategy for therapeutic brain stimulation. DBS-induced mania has been reported following stimulation of ventral capsule/ventral striatum (VC/VS) or subthalamic nucleus (STN) in patients with obsessive-compulsive disorder [130] or Parkinson’s disease [131], respectively, and has been associated to lower DBS efficacy [130, 131]. Similar to what was described for TMS and tDCS targets, we found that VC/VS [132–135] and STN [136–138] locations associated with DBS-induced mania were more connected to the lesional mania map [111] than standard DBS targets [139, 140]. Interestingly, right DBS-STN electrodes that are ventromedially located are more functional connected to right OFC and associated with disinhibited behavior, a core symptom of mania [141, 142]. Future studies should be conducted to improve the understanding of DBS-induced mania neurobiology, namely exploring which brain connectivity networks are impaired and if they truly converge with the proposed functional neuroanatomic model of mania. As in TMS and tDCS, such findings could lead to great improvements in DBS treatment planning, increasing DBS efficacy while avoiding the emergence of undesirable neuropsychiatric side effects.

### Mood laterality and hemispheric imbalance in mania

Emotions are core human experiences [32] and, as mentioned above, their neurobiology has been historically associated to lateralized brain function, in the so-called mood laterality hypothesis [80, 81]. According to this theory, negative and positive emotions are linked to the right and left brain hemisphere, respectively [80]. In part, this theory is based on the fact that left-sided brain impairment is predominantly associated with depressive episodes [94, 143–145], while manic syndromes are more associated with right-sided dysfunction [31, 83–87]. Similar polarized behavior phenotypes have also been observed in animal models with analogous lateralized brain damage [76]. Additionally, this hypothesis has also been supported by the clinical efficacy [18, 114–116, 118, 119] and the side-effects profile [118–124] observed in lateralized non-invasive brain stimulation techniques that are used to treat affective disorders [112, 113, 117]. Nevertheless, the mood laterality theory cannot be interpreted assuming an all-or-nothing rationale i.e., only left-sided brain structures are associated with positive emotions and vice-versa. Such a view not only is a clear overgeneralization [91] but may also ignore other theories of the neurobiology of emotion [146]. Instead, many authors have suggested focusing on a right-left imbalance on key brain structures [28, 81, 83, 91, 94–102], a possibility that we believe is supported by available findings on the functional neuroanatomy of mania.

While altered structure and function of the right brain have been consistently reported in mania, it does not follow a random distribution, with specific limbic regions consistently affected [16, 86, 92, 111, 147–153]. In fact, the evidence regarding brain regions affected unilaterally in both primary and lesional mania is consistent with data supporting the mood laterality hypothesis [80, 81]. The right VMPFC and VLPFC, which are key limbic hubs associated with emotional behavior regulation [32, 35], were shown to be hypoactive in mania [22, 24, 26, 27, 31, 39, 40, 46, 92, 111]. There is also evidence that right mesiotemporal structures, such as the amygdala, as well as the right basal ganglia [27, 29, 53, 92], both of which have also been associated with emotional behavior [16, 32–34], are impaired in patients with mania. On the other hand, left-sided limbic regions were shown to be overactive in mania, namely amygdala [24–26, 110], ACC [35, 40, 48, 51, 52], and basal ganglia [27, 52, 53], that are also critical regions in regulation of emotional behavior [32, 35]. Hence, when right- and left-sided findings are interpreted jointly, they suggest that, during mania, brain activity is imbalanced, towards right-sided hypoactivity and/or left-sided hyperactivity [91]. Conversely, as mentioned above, caution should be taken when considering the lateralized model of the functional neuroanatomy of mania. While consistent evidence supports such a pattern in specific regions, a complete lateralized model is unlikely. In fact, some of the presented evidence may argue for bilateral dysfunction in other key limbic regions, such as the ventromedial prefrontal cortex or basal ganglia. Future research in patients with mania specifically studying right-left activity imbalance, as well as connectivity pattern, in these or other brain regions, would help confirm or disprove the lateralization hypothesis, while further exploring the potential mechanism for its occurrence (e.g., right-left disconnection in key hubs of the limbic circuit).

### Limitations

This review has potential limitations that should be considered when interpreting its conclusions. First, rather than a systematic literature review, it is a comprehensive overview of the evidence regarding the functional neuroanatomy of mania. While a systematic literature review may better account for potential biases and limitations at the single experiment level, we have not avoided the inconsistent findings available in the literature. In fact, we offered potential explanations and/or future directions to resolve conflicting results. Moreover, this general overview, where we have summarized the best available evidence while creating a convergent model for mania circuit dysfunction, may encourage others to pursue questions that remain unsolved, further improving the quality and quantity of published evidence addressing this question. Second, most studies reviewed here have small sample sizes, creating challenges for the interpretation and generalizability of results, while underlining the need for larger sample sizes in future studies, for example using neuroimaging consortia [154–157]. In the current manuscript, we have nevertheless attempted to overcome this limitation by including results from metanalyses [22, 23] that, while focusing more broadly on bipolar disorder rather than specifically on mania, contributed towards resolving conflicting evidence at the experimental study level. Third, functional imaging studies are very heterogeneous in methodology, ranging from studies analyzing resting state to others studying responses to emotional stimuli or paradigms of cognitive control and response inhibition, limiting the possibility of global interpretation of results, and suggesting the need for standardization of study paradigms. Nonetheless, as proposed above, conflicting findings obtained in similar regions with different task paradigms may reflect the dynamics of mania neurobiology, with a specific brain region pathologically activated or deactivated depending on the context. Finally, at the individual study level, including lesion studies, there is heterogeneity or lack of specificity in terminology for brain regions, particularly in the prefrontal cortex, hindering the convergence of findings from different studies. Additionally, information is also lacking regarding lateralization of results, since the side is omitted from the description of study results in some studies. Both uniform terminology and laterality description are critical to reach a convergent model for functional neuroanatomy of mania, and future studies should consider these points when reporting their results. Here, we have addressed this limitation providing the description of each study result, while offering our interpretation of where such findings may converge in a proposed model for the functional neuroanatomy of mania.

## Conclusions

Here, we have reviewed research supporting the hypothesis that the functional neuroanatomy of mania is centered in lateralized disruption of specific regions within limbic circuits. While findings were heterogenous, as expected for a neuropsychiatric condition such as mania, the available evidence was globally consistent with the conclusion that, in primary idiopathic mania, there is reduced activity in right ventral PFC and increased activity in the left amygdala, left ACC, and left basal ganglia. Although this model is certainly an over-simplification of the functional neuroanatomy of mania, it is consistent with findings from lesional mania and therapeutic neurostimulation, suggesting that it may be an adequate approximation to the dysfunctional circuits associated with this condition. Most importantly, we expect that this model may contribute to optimize brain stimulation targets, namely in the prefrontal cortex, for future research on the treatment of mania, and to avoid mania as a side effect in the treatment of other conditions. Ultimately, exploring the potential therapeutic implications of this model may help clarify not only which regions and targets are clinically meaningful for the treatment of mania but also unipolar and bipolar depression, mixed affective states, or even bipolar disorder irrespective of the episode polarity.
